# Intense light mitigates hypoxia-induced right ventricular remodeling and dysfunction through reducing inflammation associated with PF4^+^ resident macrophages

**DOI:** 10.1016/j.gendis.2025.101867

**Published:** 2025-10-21

**Authors:** Dingyuan Tian, Yingzi Pan, Xiaoyue Lai, Xinyu Bao, Pan Zheng, Yan Tan, Chun Liu, Ziyang Wang, Qingyuan Yang, Yang Liu, Xiaoqin Wan, Zhihui Zhang, Fang Deng

**Affiliations:** a Department of Pathophysiology, College of High Altitude Military Medicine, Army Medical University, Chongqing 400038, China; b Department of Cardiovascular Medicine, Laboratory of Chronobiology and Cardiometabolic Disease, Southwest Hospital, Army Medical University, Chongqing 400038, China; c Department of Stomatology, Daping Hospital, Army Medical University, Chongqing 400038, China; d Department of Ultrasound, Xinqiao Hospital, Army Medical University, Chongqing 400038, China; e Key Laboratory of Geriatric Cardiovascular and Cerebrovascular Diseases, Ministry of Education, Chongqing 400038, China; f Key Laboratory of Extreme Environmental Medicine, Ministry of Education of China, Chongqing 400038, China

**Keywords:** Hypoxia, Intense light, Macrophages, PF4, Right ventricular remodeling

## Abstract

Hypoxia-induced right ventricular (RV) remodeling and dysfunction present a significant health risk to populations experiencing prolonged hypoxic conditions. Intense light, a noninvasive and easily implemented intervention, has previously been reported to exert cardioprotective effects by improving myocardial ischemia. However, whether intense light provides protective benefits against RV remodeling and the underlying mechanisms remain largely unexplored. In this study, we established mouse models exhibiting RV remodeling and dysfunction through long-term hypoxia to investigate the protective effects of intense light. Echocardiography, hemodynamic parameters measurements, and Fulton index assessments were employed to evaluate RV dysfunction and remodeling. Additionally, single-nuclei RNA sequencing, immunohistochemistry, immunofluorescence, and western blotting analyses were conducted to identify targeted genes in macrophage-associated inflammation within the heart. The results indicate that intense light significantly alleviates hypoxia-induced RV remodeling and dysfunction in mice. Intense light may mediate macrophage-associated inflammation through differentially expressed genes, including PF4, as well as the quantity of macrophages in the right ventricles (RVes). Resident macrophages (Res_Macro) demonstrate cardioprotective effects when intense light is applied, which mitigates RV remodeling. Our findings also suggest that PF4 expression and the presence of PF4^+^ resident macrophages (Res_PF4^+^_Macro) are linked to the attenuation of RV remodeling by intense light. Macrophage PF4 expression and the quantity of PF4^+^ macrophages in the RVes are closely associated with the levels of RV remodeling and dysfunction. This study unveils a novel noninvasive approach for the prevention of RV remodeling and dysfunction induced by hypoxia, and indicates that Res_PF4^+^_Macro and PF4 expression could be potential intervening targets.

## Introduction

Hypoxia exacerbates persistent pulmonary vasoconstriction and remodeling of the pulmonary artery. Initially, the right ventricle (RVe) compensates for increased pressure through hypertrophy; however, this adaptation ultimately progresses to right ventricular (RV) failure.^1^^,^^2^ Recent studies have highlighted that RV hypoxia plays a crucial role in both RV hypertrophy and failure associated with hypoxic pulmonary hypertension.^3^ Previous investigations into specific pathways leading to RV remodeling and dysfunction have identified various interventions targeting metabolism or angiogenesis, as well as strategies aimed at reactive oxygen species or hypoxia-inducible factor 1 (HIF-1).^4^, ^5^, ^6^ Nevertheless, these approaches have demonstrated only partial success in animal models due to issues such as lack of specificity, inadequate agents, and incomplete efficacy.^4^ Consequently, there are limited interventions available that can be broadly applied to prevent or treat hypoxia-induced RV remodeling and dysfunction.

Intense light has been recognized to promote multiple types of circadian rhythm sleep disorders, accompanied by various metabolic diseases.^7^^,^^8^ Previous studies revealed that intense light played a cardioprotective role in improving myocardial ischemia via enhancing the circadian amplitude of period circadian regulator 2 (PER2).^9^^,^^10^ Since intense light could also mitigate the inflammation under hypoxia,^11^ it holds the potential to alleviate RV remodeling and dysfunction induced by hypoxia. However, the effect of intense light on the RV and the underlying mechanism remains to be elucidated yet.

The pathogenesis of hypoxia-induced RV remodeling is complex and heterogeneous, among which cardiac inflammation plays an important role.^12^ Cardiac macrophages are nodal regulators of inflammation, leading to pressure overload-induced myocardial remodeling, including RV remodeling and failure.^13^^,^^14^ Activation and recruitment of macrophages in the heart were involved in mediating the RV remodeling.^15^ Resident macrophages in the heart play a crucial role in eliminating mitochondria and other vesicular materials secreted by cardiomyocytes, thereby preventing the accumulation of extracellular waste.^16^ Besides, resident macrophages may facilitate cardiac remodeling by interacting with adjacent cardiomyocytes in response to mechanical stretch or their own inducible depletion.^17^^,^^18^ Therefore, targeting reduced macrophage-associated inflammatory response and focusing on specific resident macrophages could be a novel approach for RV remodeling caused by hypoxia.

To this end, our study demonstrated that intense light effectively alleviated hypoxia-induced RV remodeling and dysfunction in mice. We observed that macrophage-associated inflammation in the heart was induced under hypoxic conditions; however, this inflammation could be mitigated by intense light. Furthermore, we found that intense light may suppress platelet factor 4-positive (PF4^+^) resident macrophage-related inflammation in the RVes during hypoxia. This study not only reveals a novel noninvasive preventive approach for RV remodeling and dysfunction caused by hypoxia but also elucidates PF4^+^ resident macrophages as a promising new target for intervention.

## Method and materials

### Animals

C57BL/6 J mice were obtained from the Jackson Laboratory (Bar Harbor, USA). Male mice were used in our study to eliminate the potential impacts of sex on hypoxia-induced RV remodeling. Mice were 6 weeks of age on arrival and fed in a 12-h light/12-h dark cycle for 2 weeks. Housed in a hypobaric oxygen chamber (GuiZhou FengLei Aviation Armament CO., Ltd., China), 8-week-old mice were first randomly divided into three groups and treated with normoxia (equivalently 21% O_2_) or hypoxia (equivalently 10% O_2_)^1^ under normal room light or intense light. 200 lux was set as the illuminance of room light, and 10,000 lux was figured out as a suitable illuminance level for intense light. All these mice were treated for 28 days and then subjected to echocardiographic measurements, catheter-based hemodynamic parameter measurements, and sampling. Mice were euthanized with pentobarbital sodium (150 mg/kg) and subsequently sacrificed by cervical dislocation to ensure death. These procedures mentioned above were approved by the Animal Ethics Committee of Third Military University (approval number: AMUWEC20223610).

### Echocardiographic measurements

Echocardiographic measurements were performed in mice at 12 weeks of age, using the Vevo2100 imaging system (FUJIFILM VisualSonics, Canada) with an MS-400 probe. Mice were anesthetized using 3% isoflurane in the chamber and underwent continuous gaseous anesthesia with 1% isoflurane by a small animal anesthesia machine (R500, RWD, China). A parasternal long-axis view of the left ventricle (LV) was obtained to evaluate its function, including parameters such as ejection fraction (EF) and fractional shortening (FS). Pulsed Doppler imaging was used to acquire the pulmonary artery spectrum to measure pulmonary artery acceleration time/pulmonary artery ejection time (AT/ET) to indicate pulmonary hypertension. RV function was evaluated by tricuspid annular plane systolic excursion (TAPSE), RV peak systolic myocardial velocity (s'), and fractional area change (FAC). A left ventricular short-axis view at the section of papillary muscles was obtained to measure RV free wall thickness (RVFW), RV end-diastolic inner dimension (RVDD), and FAC. A focused right-sided heart apical 4-chamber view was acquired to assess the levels of TAPSE and s' at the tricuspid annulus of the RV free wall. The TAPSE level was assessed using M-mode as the movement of the tricuspid annulus from end systole to end diastole. The s' level was measured as the peak systolic velocity by tissue Doppler imaging.

### Catheter-based hemodynamic parameter measurements

To investigate the level of pulmonary artery pressure, we assessed the RV systolic pressure (RVSP) of mice anesthetized with 1% pentobarbital sodium. A 1.2-F pressure catheter (SciSense Inc., Ontario, Canada) was inserted into the right jugular vein and advanced into the RVes to measure RVSP. All data were analyzed in a blinded manner using the PowerLab data acquisition system (AD Instruments, Bella Vista, Australia) and averaged over ten sequential beats.

### Heart sampling

The hearts of mice were sampled and weighed. To quantify the RV hypertrophy, the free wall of the RVe and the left ventricle plus septum (LV + S) were dissected and weighed, and the Fulton index was calculated using the formula [RV/(LV + S)]. Half of the RVes were fixed in 4% paraformaldehyde (pH = 7.4) at 4 °C for 24 h, gradiently dehydrated with alcohol at the doses of 70%, 75%, 80%, 85%, 90%, 95%, and 100%, and transparented by xylene. These samples were fabricated as paraffin tissue blocks. Others were snap-frozen in liquid nitrogen and stored at −80 °C for single-nucleotide RNA sequencing (snRNA-seq) or future biochemical analysis.

### Cell preparation

After being harvested, the RVes were washed in ice-cold RPMI1640 and dissociated using Tissue Dissociation Reagent A (Seekone K01301-30) from SeekGene according to instructions. Cell count and viability were estimated using a fluorescence cell analyzer (Countstar® Rigel S2) with AO/PI reagent after removal of erythrocytes (Solarbio R1010). Finally, fresh cells were resuspended at 1 × 10^6^ cells per mL in 1 × phosphate-buffered saline and 0.04% bovine serum albumin.

### snRNA-seq library construction and sequencing

The RVes of mice were subjected to snRNA-seq. snRNA-seq libraries were prepared using SeekOne® Digital Droplet Single Cell 3′ library preparation kit (SeekGene Catalog No. K00202). Briefly, cell nuclei were mixed with reverse transcription reagent and then added to the sample well in SeekOne® DD Chip S3. Barcoded hydrogel beads and partitioning oil were dispensed into chip S3. Subsequently, reverse transcription was performed at 42 °C for 90 min and inactivated at 80 °C for 15 min. Next, cDNA was purified from the broken droplet and amplified in a PCR reaction. Finally, the indexed PCR was performed to amplify the DNA representing the 3′ polyA part of the expressed genes. The indexed sequencing libraries were subjected to clean-up with SPRI beads, quantified by quantitative PCR (KAPA Biosystems KK4824), and then sequenced on Illumina NovaSeq 6000 with PE150 read length.

### snRNA-seq data processing

We first processed the raw sequencing data by fastp^19^ and then used SeekSoul Tools (version 1.0.0) to process the sequence data and aligned it to mouse (mm10) to obtain a gene expression matrix. We used Seurat (version 4.0.0) to filter low-quality cells, omitted cells with a number of detected genes <200, and used median absolute deviation-variance normal distribution to remove cells affected by mitochondrial genes.^20^

### Canonical correlation analysis, dimensionality reduction, and clustering

After quality control and filtering, library-size normalization to each cell was performed by NormalizeData of Seurat (version 4.0.0).^21^ Then, the RunPCA and RunUMAP were used to reduce dimensions. FindClusters was used to cluster cells using the 30 dims at a resolution of 0.6.

### Differentially expressed gene (DEG) analysis

The FindMarkers function was used to identify DEGs. Genes were ranked by absolute log_2_ fold-change (log_2_FC), and those with *P* values > 0.05 (adjusted for multiple comparisons) and log_2_FC < 0.25 were removed.

### Enrichment analysis

Gene Ontology (GO) enrichment analysis of DEGs was implemented by the clusterProfiler R package (version 4.10.0).^22^ GO terms with corrected *P*-value less than 0.05 were considered significantly enriched by DEGs. Kyoto Encyclopedia of Genes and Genomes (KEGG) was a database resource for understanding high-level functions and utilities of the biological system (http://www.genome.jp/kegg/). We used the clusterProfiler R package to test the statistical enrichment of DEGs in KEGG pathways.

### Single-cell trajectory analysis

The Monocle2 package (version 2.26.0) was used to determine the potential lineage differentiation.^23^ The DDRTree method was utilized for dimension reduction and cell ordering along the pseudotime trajectory. No root state was specified. Branch analysis was performed with the BEAM function. The significantly changed genes in the branch point were clustered using plot_genes_branched_pseudotime functions according to the distinct patterns of gene expression change.

### Immunohistochemical and immunofluorescence analysis

A series of 5-μm sections of the RVes of mice were subjected to 0.05% Triton and 0.03% H_2_O_2_ for 20 min, 3% bovine serum albumin for 30 min, and then stained with a CD68 antibody (E3O7V, Cell Signaling Technology, UK) or a Col1A antibody (EPR24331-53, Abcam, UK) at 4 °C overnight. After washing with phosphate-buffered saline, goat anti-rabbit IgG biotinylated secondary antibody (BA-1000, Vector, Germany) and streptavidin (SA-5704, Vector, Germany) were added for further incubation. ImmPACT DAB substrate (SK-4105, Vector, Germany) was used for a chromogenic reaction. The presence of PF4 and resident macrophages in the heart were detected by double immunofluorescence staining with a PF4 antibody (A3694, Abclonal, China) and a Lyve1 antibody (14-0443-82, ThermoFisher, USA). The donkey anti-rat antibody (ab150156, Abcam, UK) and anti-rabbit antibody (4412S, Cell Signaling Technology, UK) were incubated as secondary antibodies. Images were acquired on a laser confocal microscope (ZEISS, Germany) and processed using Image J software in a blinded manner.

### Western blotting

Proteins were extracted from the homogenates of the RVes in RIPA lysis buffer (89900, Thermo Scientific, USA) with protease and phosphatase inhibitors (04693124001, 04906837001, Roche, USA). The samples were separated on SDS-PAGE gels (4%–20% acrylamide, Genscript, USA) and transferred to polyvinylidene fluoride membranes (Cytiva, USA). The blots were probed with the following primary antibodies: PF4 antibody (sc-398979, Santa cluz, USA), tumor necrosis factor-alpha (TNF-α) antibody (A24214, Abclonal, China), interleukin-6 (IL-6) antibody (A0286, Abclonal, China), and anti-β-actin antibody (PTM-5028, PTM Bio, China). The membranes were incubated with horseradish peroxidase-conjugated secondary antibodies (HA1001, HUABIO, China) and scanned with ChemiDoc Imaging Systems (BIO-RAD, USA). The band density was normalized to that of β-actin for analysis.

### Statistical analysis

The data were presented as mean ± standard error of the mean. Analyses of RV dysfunction and remodeling were conducted using SPSS version 25.0 (Chicago, USA), while figures were generated with GraphPad Prism software version 8.0 (San Diego, USA) and Adobe Illustrator (California, USA). Normality and homogeneity of variance tests were performed for these assays. Comparisons among the three groups were carried out using one-way *ANOVA* followed by Tukey's post hoc test. A *P*-value less than 0.05 was deemed statistically significant. All analyses in this study were performed with blind evaluation.

## Results

### Intense light alleviates hypoxia-induced RV dysfunction and RV remodeling

**Figure 1:**
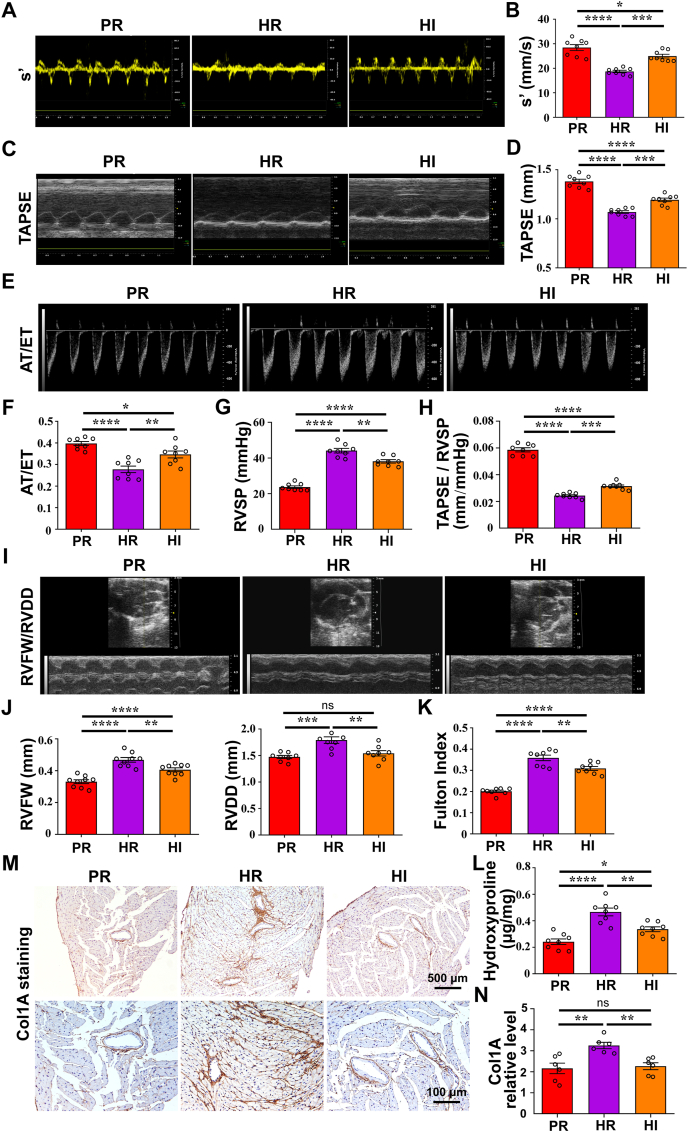
The levels of RV hypertrophy and RV dysfunction in mice subjected to normoxia or hypoxia with room light and hypoxia with intense light. **(A**–**D)** The s' and TAPSE levels of mice in each group and the statistical analyses. **(E, F)** The AT/ET levels of mice in each group and the statistical analyses. **(G)** The levels of RVSP in mice of each group. **(H)** The levels of TAPSE/RVSP in mice of each group. **(I, J)** The RVFW and RVAW levels and the statistical analyses. **(K)** The levels of RV hypertrophy (Fulton index). **(L)** The level of hydroxyproline in the RVes. **(M, N)** The levels of Col1A in the RVes. *n* = 8 per group. ∗*P* < 0.05; ∗∗*P* < 0.01; ∗∗∗*P* < 0.001; ∗∗∗∗*P* < 0.001; ns, no significant. The error bars represent the standard error of the mean. RVe, right ventricle; RV, right ventricular; HR, hypoxia and room light; PR, physiological normoxia and room light; HI, intense light and hypoxia; s', RV peak systolic myocardial velocity; TAPSE, Tricuspid annular plane systolic excursion; AT/ET, pulmonary artery acceleration time/pulmonary artery ejection time; RVSP, RV systolic pressure; RVFW, RV free wall thickness; RVDD, RV end-diastolic inner dimension; Col1A, collagen type I alpha 1.

To investigate whether intense light alleviated RV dysfunction induced by hypoxia, echocardiographic measurements and hemodynamic parameter measurements were performed. The levels of RV systolic function indicators, including s', TAPSE, and FAC, were significantly lower in the hypoxia/room light (HR) group compared with those the physiological normoxia/room light (PR) group that were almost identical to those of the intense light (PI) group (Fig. S1A–C). PR was designated to represent the control in subsequent experiments. These indicators in the mice subjected to intense light and hypoxia (HI) were found to be higher than those in the HR group (Fig. 1A–D; Fig. S2A, B). Compared with PR mice, HR mice showed increased AT/ET levels, which were attenuated in HI mice (Fig. 1E, F). Consistently, the level of RVSP was also found down-regulated in HR mice and up-regulated in HI mice (Fig. 1G), indicating that there exists a higher level of pulmonary artery pressure in HR mice and a lower level in HI mice. Intense light also improved the decreased level of TAPSE/RVSP under hypoxia, which reveals the improved efficiency of the RVes in overcoming pulmonary artery resistance (Fig. 1H). The reduced level of pulmonary hypertension and the heightened level of RV function observed in the HI group, as compared with the HR group, suggested that intense light not only mitigates the pulmonary artery pressure level and RV afterload but also alleviates the hypoxia-induced RV dysfunction.

Reduced EF and FS levels were also observed in the HR group compared with the PR group, while increased EF and FS levels were noted in the HI group compared with the HR group (Fig. S2C, D). Intense light and hypoxia also impacted LV function under the 4-week intervention. Besides, HR mice exhibited lower heart rate levels than PR mice (Fig. S2E). No significance in the heart rate was observed between the HR and HI groups, indicating that intense light did not influence heart rate. Thus, intense light also attenuated hypoxia-induced reduced LV function levels. We also observed that the levels of RVFW, RVDD, and RV hypertrophy (the Fulton index) were significantly elevated in the HR group versus the PR group. Compared with the HR group, the HI group exhibited significantly lower levels of RVFW, RVDD, and RV hypertrophy (Fig. 1I–K). These results suggest that intense light could alleviate RV hypertrophy and dilation under hypoxia. Besides, we performed collagen type I alpha 1 (Col1A) staining and hydroxyproline level detection on the RVes. Intense light intervention was also observed to ameliorate cardiac fibrosis in the RVes under hypoxia (Fig. 1L–O). These findings revealed that hypoxia leads to RV remodeling, while intense light intervention has the potential to mitigate this hypoxia-induced RV remodeling.

### Single-cell characterization of RV remodeling in response to hypoxia and/or intense light

**Figure 2:**
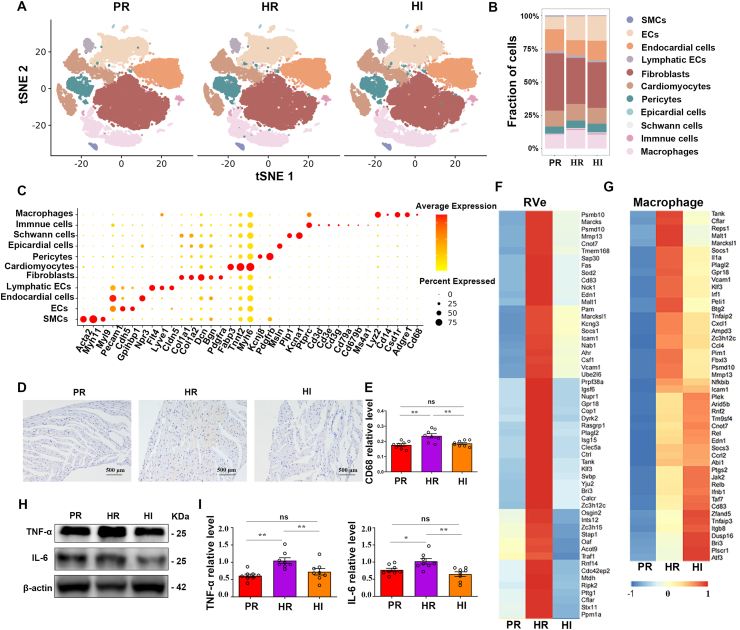
Single-cell profiles of the RVes and macrophage-associated inflammation in the PR, HI, and HR groups. **(A)** The t-distributed stochastic neighbor embedding (t-SNE) analysis of cell types in the RVes of each group. Each dot represents an individual cell, with colors denoting the specific cluster to which it is assigned. *n* = 3 per group. **(B)** The percentage bar charts visualize the proportion of cell types in each group. *n* = 3 per group. **(C)** The representative gene expression of different cell types in the RVe. *n* = 3 per group. **(D, E)** The expression of CD68 in the RVes of mice and the statistical analyses. *n* = 6 per group. **(F, G)** The heatmap of inflammation-related genes in all cell subsets (left) or macrophages (right). *n* = 3 per group. **(H, I)** The expression of TNF-α and IL-6 in the RVes and the statistical analyses. *n* = 6 per group. ∗*P* < 0.05; ∗∗*P* < 0.01; ns, no significant. The error bars represent the standard error of the mean. RVes, right ventricles; HR, hypoxia and room light; PR, physiological normoxia and room light; HI, intense light and hypoxia; SMC, smooth muscle cell; EC, endothelial cell; CD68, cluster of differentiation 68; TNF-α, tumor necrosis factor-alpha; IL-6, interleukin-6.

Concerning the unknown underlying mechanisms of intense light alleviating RV dysfunction and remodeling under hypoxia, we reconstructed the single–cell profiles of the RVes in each group. Eleven cellular subtypes were identified, including smooth muscle cells, endothelial cells, endocardial cells, fibroblasts, cardiomyocytes, and macrophages (Fig. 2A). Compared with the PR group, the proportion of macrophages was elevated in the HR group, while it decreased in the HI group compared with the HR group (Fig. 2B). Moreover, we presented the expression of certain specific genes of different cell subtypes, highlighting the substantial heterogeneity among the cellular subsets (Fig. 2C; Fig. S3A–C). Correspondingly, the immunohistochemical assay showed that the CD68 expression level was higher in the HR group compared with the PR group. HI mice showed reduced CD68 expression level compared with HR mice (Fig. 2D, E). These findings indicate that the number of macrophages in the RVe significantly increased under hypoxia and decreased with intense light prevention.

Given the role of inflammation played in RV dysfunction and remodeling under hypoxia and intense light, we analyzed the expression of inflammation-related genes in the RVes and macrophages. We found an activation of pro-inflammatory factors in the HR group and a reduction in the HI group in the RVes and macrophages (Fig. 2F, G). It suggests that there is a remarkable macrophage-associated pro-inflammatory shift mediated by intense light. Consistently, the levels of key macrophage-related cardiac inflammatory factors, such as TNF-α and IL-6, were increased in the RVes of HR mice while decreased in HI mice (Fig. 2H, I). These findings suggested that macrophage-associated inflammatory response in the RVes induced by hypoxia could be attenuated by intense light.

### Intense light attenuating inflammation activation is associated with PF4 expression and macrophage subset specificity

**Figure 3:**
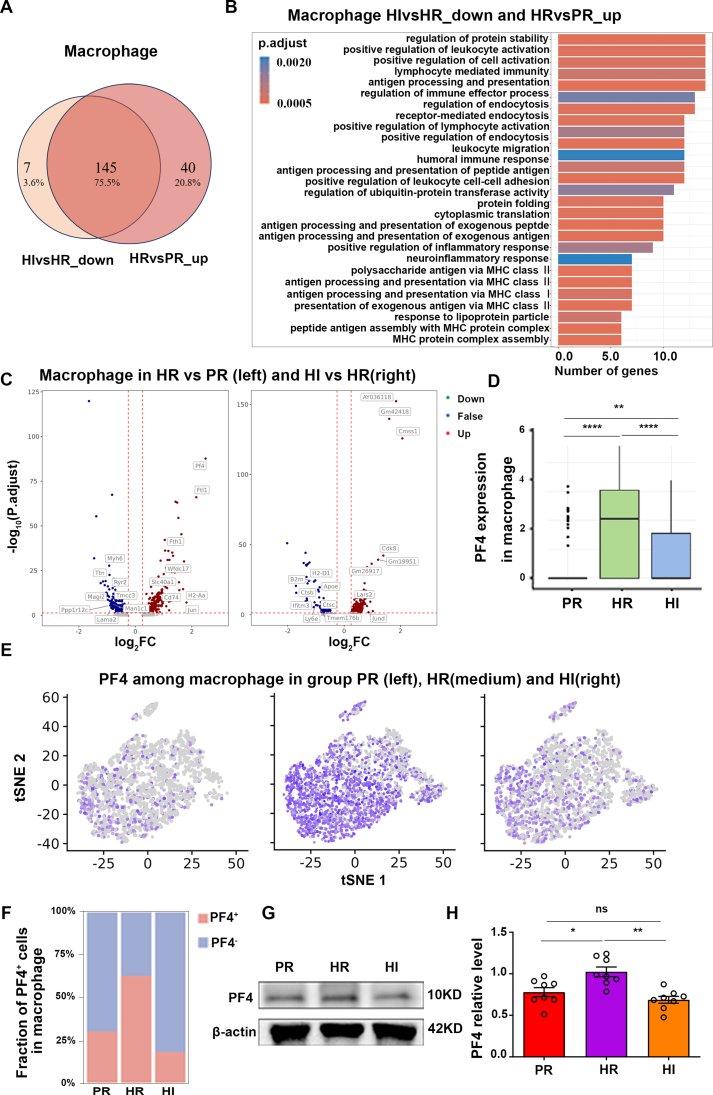
PF4 expression and PF4^+^ macrophages played an important role in intense light attenuating cardiac inflammation under hypoxia. **(A, B)** The number of DEGs and enrichment pathways in the macrophages between the HI and HR groups and between the HR and PR groups. *n* = 3 per group. **(C)** DEGs in the macrophages between the HI and HR groups and between the HR and PR groups. *n* = 3 per group. **(D)** The PF4 expression in the macrophages of the RVes in each group. *n* = 3 per group. **(E)** The t-distributed stochastic neighbor embedding (t-SNE) map of PF4 expression in the macrophage subset of the RV. *n* = 3 per group. **(F)** The proportion of PF4^+^_Macro and PF4^−^_Macro of the RVes. *n* = 3 per group. **(G, H)** The PF4 expression in the RVes and the statistical analyses. *n* = 6 per group. ∗*P* < 0.05; ∗∗*P* < 0.01; ∗∗∗∗*P* < 0.0001; ns, no significant. The error bars represent the standard error of the mean. DEG, differentially expressed gene; RVes, right ventricle; HR, hypoxia and room light; PR, physiological normoxia and room light; HI, intense light and hypoxia. PF4, platelet factor 4.

DEGs in the macrophages of the RVes in different groups were further analyzed. One hundred and forty-five genes notably were down-regulated in the HI group versus the HR group (HI *vs*. HR_down) and were found to overlap with those that were up-regulated in HR mice versus PR mice (HR *vs*. PR_up), such as β2-microglobulin (B2m) and serpin family H member 1 (Serpinh1) (Fig. 3A and Table S1). For instance, it is well known that B2m typically binds to major histocompatibility complex class 1 (MHC I) molecules and participates in the antigen presentation process.^24^ Intense light that mitigates hypoxia-induced RV remodeling shares a significant number of co-regulated genes with those involved in hypoxia-induced RV damage. GO functional analysis also presented that macrophage activation and inflammation-associated processes were enriched, such as regulation of endocytosis and antigen presentation (Fig. 3B). To explore the key targeted genes associated with macrophage activation, a pairwise comparison of DEGs in the macrophages was conducted. Compared with the PR group, PF4 expression was significantly up-regulated in the HR group. Whereas in the HI group, PF4 expression was down-regulated compared with the HR group (Fig. 3C). This suggests that PF4 expression may play a crucial role in the improvement of hypoxia-induced RV dysfunction and remodeling by intense light. We visualized the effect of PF4 on macrophages (Fig. 3D) and divided the macrophages into PF4-positive (PF4^+^) or PF4-negative (PF4^−^) macrophages. The quantity of PF4^+^ macrophages in the RVes was significantly higher in the HR group compared with the PR group, whereas it was notably lower in the HI group than in the HR group (Fig. 3E, F). Consistently, the protein expression level of PF4 in the RVes was higher in the HR group and lower in the HI group (Fig. 3G, H). It is suggested that PF4 expression and PF4^+^ macrophages exert a diverse effect on cardiac inflammation in the RVes under intense light or hypoxia.

### Resident macrophages could participate in intense light mitigating cardiac inflammation induced by hypoxia

**Figure 4:**
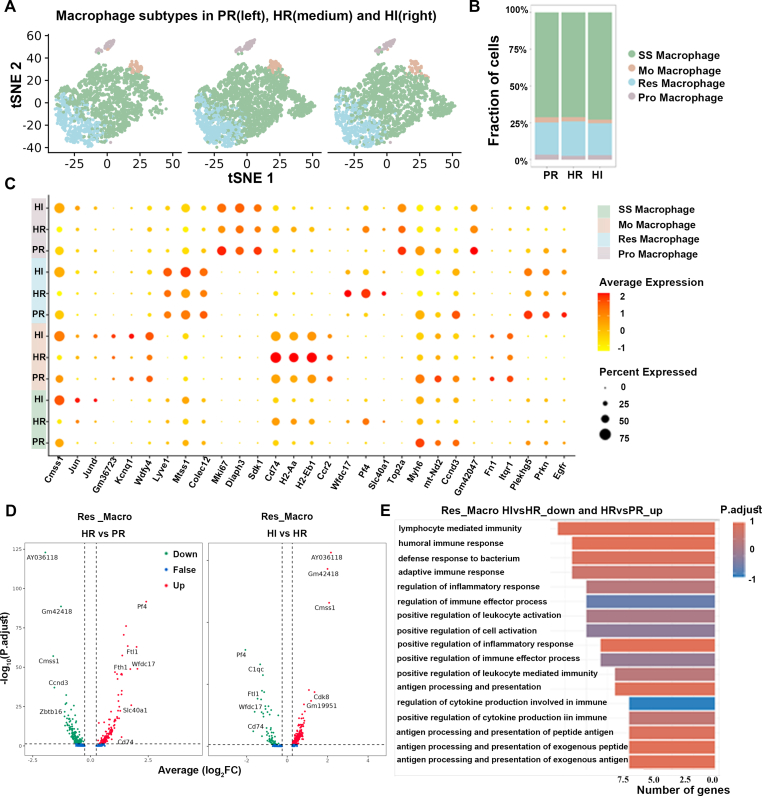
Resident macrophages exert critical effects on intense light attenuating cardiac inflammation. **(A)** The split t-distributed stochastic neighbor embedding (t-SNE) map of four macrophage subsets in the RVes. **(B)** The cell proportions of macrophage subsets in the RVes. **(C)** The heatmap of the top DEGs based on group and macrophage subsets. **(D)** The top DEGs down-regulated in HI versus HR and up-regulated in HR versus PR of Res_Macro. **(E)** GO enrichment analysis of the DEGs in (D). *n* = 3 per group. DEG, differentially expressed gene; RVes, right ventricle; HR, hypoxia and room light; PR, physiological normoxia and room light; HI, intense light and hypoxia; SS_Macro, steady-state macrophage; Mo_Macro, monocyte-derived macrophage; Res_Macro, resident macrophage; Pro_Macro, proliferating macrophage.

Through conducting a clustering analysis on macrophages, we identified four subgroups, including steady-state macrophages (SS_Macro), monocyte-derived macrophages (Mo_Macro), resident macrophages (Res_Macro), and proliferating macrophages (Pro_Macro) (Fig. 4A). We found that compared with the PR group, the proportion of Res_Macro increased in the HR group and decreased in the HI group (Fig. 4B). These results suggest that the Res_Macro subset may be a key player in the macrophages. Using the traditional M1 and M2 polarization types to classify (Fig. S4A–C), differences in the proportions of inflammation-associated macrophage subtypes among groups were not observed. However, PF4 expression in macrophage subtypes exhibited consistent differences across groups (Fig. S4D–F). We further delineated the relationship among four macrophage subsets, creating a heatmap representing the changes in key genes determined by these two variables (Fig. 4C). Consequently, we figured out that the DEGs of the Res_Macro were consistent with the variations observed in the macrophages. PF4 expression level was significantly up-regulated in the HR group while down-regulated in the PR group and the HI group (Fig. 4D). GO analysis on the Res_Macro subset revealed that genes up-regulated in the HR group compared with the PR group and down-regulated in the HI group were primarily involved in macrophage-associated inflammation pathways (Fig. 4E and Table S2). These findings demonstrated that intense light might inhibit the inflammatory response under hypoxia through the Res_Macro and PF4 expression.

### PF4 expression and Res_PF4^+^_Macro are associated with intense light-attenuating cardiac inflammation

**Figure 5:**
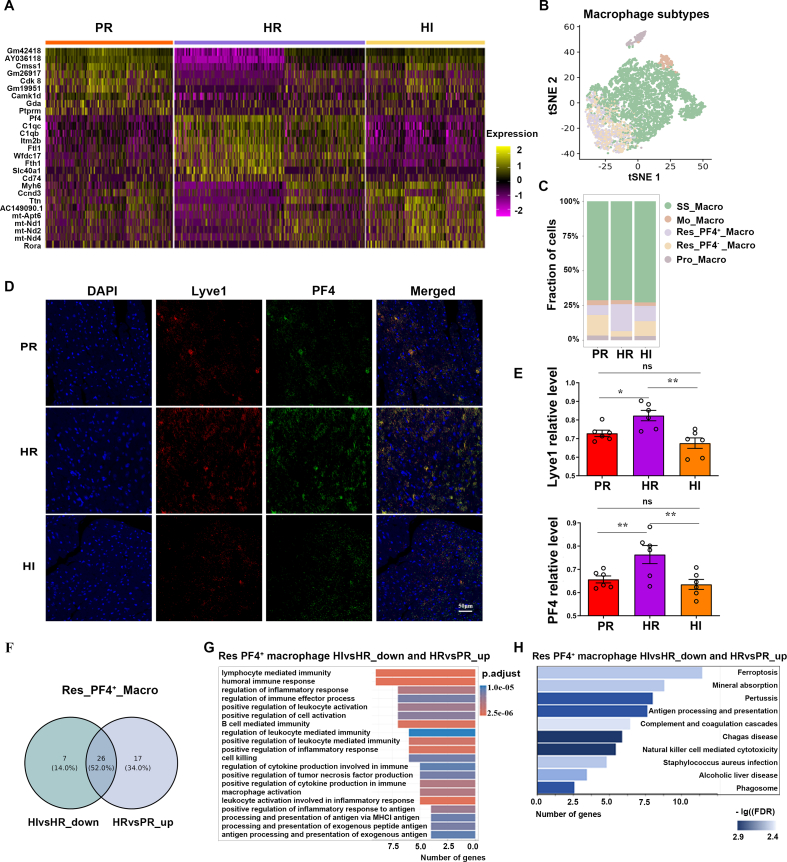
PF4 mediates the effect of resident macrophages on inflammation in the RV. **(A)** Top DEGs in the Res_Macro of the RV in each group. *n* = 3 per group. **(B)** The split t-distributed stochastic neighbor embedding (t-SNE) map of five macrophage subsets in the RV. *n* = 3 per group. **(C)** The proportions of macrophage subsets in the RV. *n* = 3 per group. **(D, E)** Detection of Res_PF4^+^_Macro in the RV and the statistical analyses. *n* = 6 per group. **(F)** The DEGs down-regulated in HI versus HR and up-regulated in HR versus PR in the Res_PF4^+^_Macro subset. *n* = 3 per group. **(G, H)** GO enrichment and KEGG pathway analysis of the DEGs identified in (F). *n* = 3 per group. ∗*P* < 0.05; ∗∗*P* < 0.01; ns, no significant. The error bars represent the standard error of the mean. PF4, platelet factor 4; DEG, differentially expressed gene; RV, right ventricles; HR, hypoxia and room light; PR, physiological normoxia and room light; HI, intense light and hypoxia; SS_Macro, steady-state macrophage; Mo_Macro, monocyte-derived macrophage; Res_Macro, resident macrophage; Pro_Macro, proliferating macrophage; Res_PF4^+^_Macro, PF4^+^ resident macrophages; Res_PF4^−^_Macro, PF4^−^ resident macrophage.

To investigate whether the protective effect of Res_Macro was mediated through PF4 expression, a detailed gene heatmap analysis of the Res_Macro subset was conducted (Fig. 5A). Among the DEGs, PF4 expression was the most significantly altered by hypoxia and intense light. It was likely that the protective effect of intense light on inflammation was mediated by PF4 expression in the Res_Macro. Resident macrophages were further subdivided into Res_PF4^+^_Macro and Res_PF4^−^_Macro (Fig. 5B). Res_PF4^+^_Macro exhibited a remarkable increase in quantity in the HR group, compared with the PR group and the HI group (Fig. 5C–E). These results imply that Res_PF4^+^_Macro may contribute to intense light against hypoxia-induced inflammation. We identified an overlap of 26 genes in the Res_PF4^+^_Macro that were up-regulated in HR mice relative to PR mice and down-regulated in HI mice relative to HR mice, including PF4 and cystatin C (Cst3) (Fig. 5F and Table S3). The knockout of PF4 has been reported to be associated with a reduction in macrophage-related recruitment.^25^ Once Cst3 has been identified in the macrophages related to immunotherapy response prediction,^26^ the effect was not elucidated. Further GO and KEGG pathway analysis presented their involvement in inflammation response and macrophage activation (Fig. 5G, H), aligning with the main function of Res_Macro in the heart. These findings reveal that PF4 expression in the Res_Macro and Res_PF4^+^_Macro may share critical inflammation-regulating roles under hypoxia in cardiac inflammation.

### The dynamic transition of Res_PF4^+^_Macro could be mediated by intense light in hypoxia-induced RV remodeling

**Figure 6:**
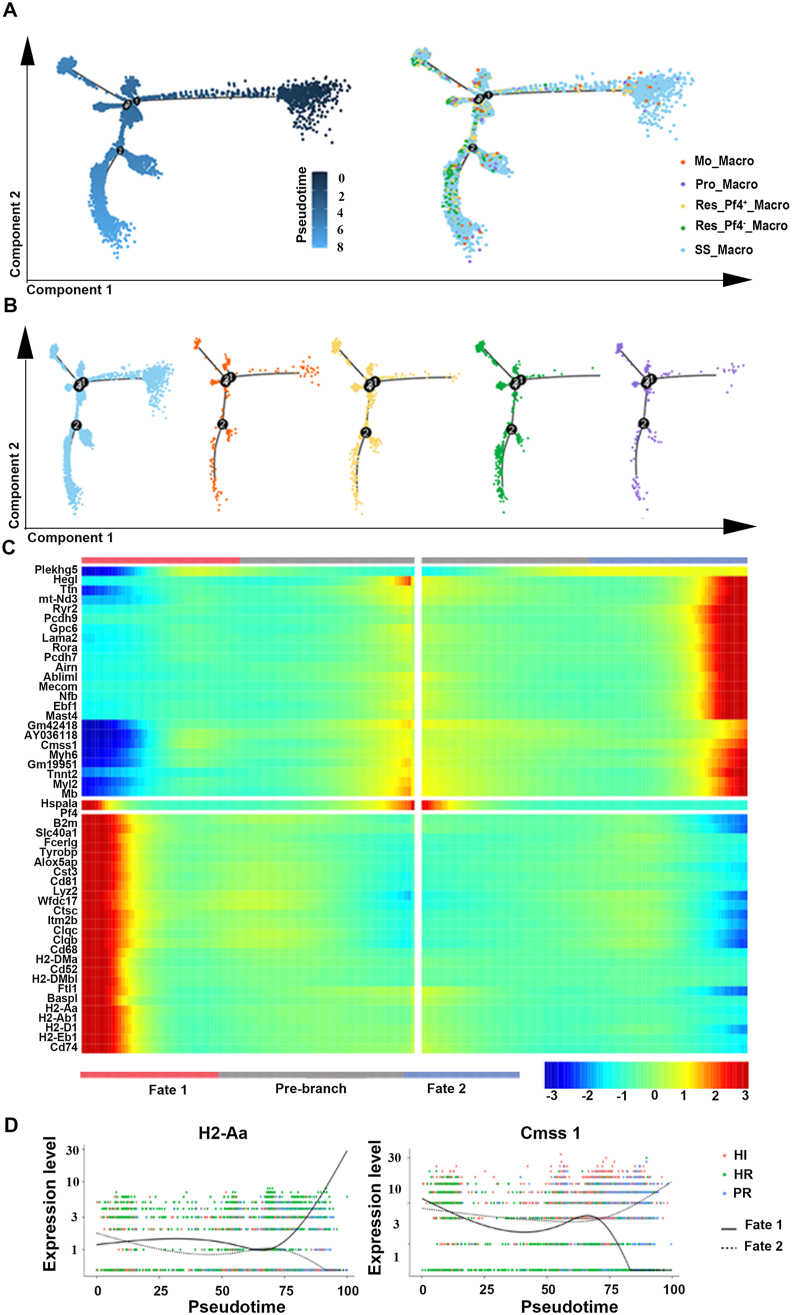
The dynamic transition of PF4-associated resident macrophages of the RVes in each group. **(A)** The Monocle prediction of macrophage differentiation and development trajectories based on Seurat clustering information. **(B)** The Monocle prediction of differentiation trajectories of each macrophage subpopulation. Each dot represents a cell, different colors represent different samples, and the numbers on the black dots merely represent nodes and have no practical significance. **(C)** The heatmap of the top 50 DEGs along with the pseudotime. The potential fate of individual subsets across pseudotime is illustrated below. Gray represents the starting position of differentiation, namely a branch (State) near the branch node and the developmental origin, namely the branch with small pseudotime; cell fate 1 represents branch node 1, corresponding to branch with small State value, and cell fate 2 represents branch with large State value for analysis node 2. **(D)** The representative contour plots showing the expression change of the gene Cmss1 and H2-Aa across pseudotime. *n* = 3 per group. RVes, right ventricle; HR, hypoxia and room light; PR, physiological normoxia and room light; HI, intense light and hypoxia; DEG, differentially expressed gene; SS_Macro, steady-state macrophage; Mo_Macro, monocyte-derived macrophage; Res_Macro, resident macrophage; Pro_Macro, proliferating macrophage; Res_PF4^+^_Macro, PF4^+^ resident macrophages; Res_PF4^−^_Macro, PF4^−^ resident macrophage. PF4, platelet factor 4; H2-Aa, histocompatibility 2 class II antigen A; Cmss1, Cms1 ribosomal small subunit homolog.

To investigate the potential transitions among macrophage subsets, we employed a pseudo-temporal analysis approach to figure out the immune states and interconversion pathways among five macrophage subtypes (Fig. 6A). We noted that SS_Macro cells were uniformly distributed across the entire pseudo-time trajectory. In contrast, Mo_Macro, Pro_Macro, Res_PF4^+^_Macro and Res_PF4^-^_Macro were present in limited numbers at the onset of differentiation. Furthermore, Res_Macro emerged as the predominant terminal state across all differentiation pathways (Fig. 6B). The heatmap of the top 50 DEGs in the pseudo-temporal sequence supports this notion (Fig. 6C), which suggest that PF4 may play a pivotal role in the inflammatory response associated with RV remodeling, as it might mediate the change of Res_PF4^+^_Macro towards Res_PF4^-^_Macro. As the PF4 and WAP four-disulfide core domain 17 (Wfdc17) expression were increased over time, the pathway shifted towards a hypoxia-related inflammatory outcome named “Fate 1” (Fig. S5A, B). We identified several gene expression changes in the pseudo-temporal analysis, including histocompatibility 2 class II antigen A (H2-Aa), Cms1 ribosomal small subunit homolog (Cmss1), and proto-oncogenes Fos and Jun (Fig. 6D; Fig. S5C). Among them, the expression of genes including PF4, H2-Aa, and Cmss1 changed, which implies that they act in coordination to mediate the transition from Res_PF4^+^_Macro to Res_PF4^−^_Macro, attenuating hypoxia-induced inflammation of RV remodeling with intense light. These findings underscore the dynamic nature of macrophage differentiation and the potential regulatory roles of specific genes in this process.

### Macrophage PF4 expression and the quantity of PF4^+^ macrophages could be potential targets of hypoxia-induced RV dysfunction and remodeling

**Figure 7:**
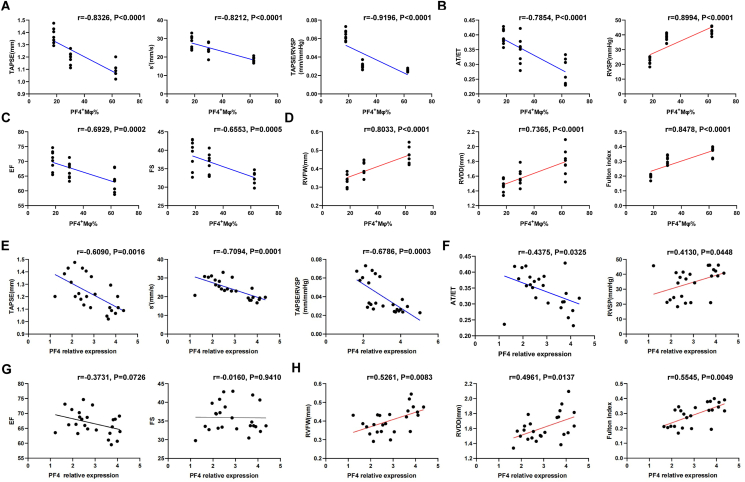
The correlation between macrophage PF4 expression or the quantity of PF4^+^ macrophages and cardiac function or RV remodeling. **(A)** The correlation between the levels of macrophage PF4 expression and RV function. **(B)** The correlation between the levels of macrophage PF4 expression and pulmonary hypertension. **(C)** The correlation between the levels of macrophage PF4 expression and LV function. **(D)** The correlation between the levels of macrophage PF4 expression and RV remodeling. **(E)** The correlation between the quantity of the PF4^+^ macrophages and RV function. **(F)** The correlation between the quantity of the PF4^+^ macrophages and pulmonary hypertension. **(G)** The correlation between the quantity of the PF4^+^ macrophages and LV function indicators. **(H)** The correlation between the quantity of the PF4^+^ macrophages and RV remodeling. *n* = 24. PF4, platelet factor 4; RV, right ventricle; LV, left ventricle.

We performed correlation analyses to ascertain if the quantity of PF4^+^ macrophages and macrophage PF4 expression in the RVes related to cardiac function and RV remodeling. The number of PF4^+^ macrophages in the RVes was significantly correlated with RV function (TAPSE, s', and TAPSE/RVSP), LV function (EF and FS), pulmonary artery pressure levels (RVSP and AT/ET), and RV remodeling (RVFW, RVDD, and Fulton index) (Fig. 7A–D). Moreover, the expression level of PF4 in the cardiac macrophages was significantly related to the levels of RV function, pulmonary artery pressure, and RV remodeling, but no correlation between the levels of macrophage PF4 expression and LV function was observed (Fig. 7E–H). The quantity of PF4^+^ macrophages exhibited a greater sensitivity to the severity of the phenotype, whereas macrophage PF4 expression level was more specifically associated with RV function and remodeling. These findings highlighted the importance of PF4 expression level in the macrophages and the quantity of PF4^+^ macrophages for diagnosing and intervening in RV dysfunction and remodeling.

## Discussion

RV hypoxia played a pivotal role in RV hypertrophy and failure during hypoxia-induced pulmonary hypertension.^3^ The onset of hypoxia-induced RV hypertrophy and failure was high; however, there exist limited effective interventions.^4^ Some studies have focused on targeted drugs for pulmonary arterial hypertension, while recent studies reported that cell-based therapies could be beneficial to the RVes by improving the microvascular system.^27^ Nevertheless, extensively effective therapies targeted at the RV remodeling and dysfunction are still lacking. Intense light has been recognized as a noninvasive and easily implemented intervention widely utilized in various metabolic diseases.^28^ The recent discovery of the beneficial effects of intense light on myocardial ischemia^29^^,^^30^ has raised questions about its potential protective role against RV remodeling and dysfunction induced by hypoxia. Our study demonstrated that intense light significantly ameliorated RV remodeling and dysfunction in mice subjected to prolonged hypoxic conditions. Furthermore, we identified an optimal illuminance level of 10,000 lux, consistent with the effective light intensity of previous studies,^30^^,^^31^ which also had enough security for future clinical applications.

Macrophage-associated inflammation in the heart could play an important role in hypoxia-induced RV remodeling.^14^^,^^32^ Though recent studies have reported that the population and activation of macrophages in the RV can induce cardiac remodeling mediated by hypoxia, the specific mechanisms underlying macrophage function in the RVes remain largely unexplored. Through snRNA-seq, we observed a significant increase in both the proportion and populations of macrophages within the RVes. Furthermore, genes and pathways associated with macrophage activation-associated inflammation were found to be up-regulated in HR mice while down-regulated in HI mice. Our findings underscore that intense light mitigates hypoxia-induced inflammation associated with macrophages in the heart.

Coded for a member of the CXC chemokine family, PF4 has been identified as an inhibitor of hematopoiesis and angiogenesis.^33^^,^^34^ Since the PF4 expression of macrophages and PF4-induced macrophages were pivotal to atherosclerosis via multiple mechanisms, including inflammation activation,^35^ they warrant further investigation in the context of hypoxia-induced RV remodeling. Interestingly, we observed that both the proportion of PF4^+^ macrophages and PF4 expression levels in the RVes were significantly altered by hypoxia and intense light exposure oppositely. On the one hand, PF4^+^ macrophages are prompt to be the key target for intense light to ameliorate RV remodeling. On the other hand, it is elucidated that PF4^+^ macrophages could represent a novel target for hypoxia-mediated RV remodeling. Moreover, resident macrophages participate in protecting from multiple cardiac diseases via regulating inflammation.^36^^,^^37^ During myocardial ischemia, resident macrophages also orchestrate inflammatory responses, improving cardiac remodeling.^38^ Analogously, resident macrophages in the RVes, especially PF4^+^ resident macrophages, were regulated by intense light via the dual-phase activity.

The expression of H2-Aa, H2-Ab1, and H2-Eb1 has been recognized to contribute to the MHC II class protein complex, related to antigen processing and exogenous peptide antigen presentation.^39^ Given that the expression of these genes in the Res_Macro could be regulated by hypoxia and intense light and were involved in the inflammation response and macrophage activation, we speculated that the expression of H2-Aa, H2-Ab1, and H2-Eb1 played an important role in the PF4^+^_Res_Macro through inflammatory regulation in the RVes. Cmss1 has been previously reported to be related to B cell-associated immunity,^40^ but its role in macrophages has not been clarified. Intense light and hypoxia oppositely influenced the expression of the Cmss1 in the Res_Macro in the RVes. Combined with consistent results observed in the pseudo-time sequence analysis, Cmss1 could play an important role in macrophage activation in the RVes under hypoxia.

This study also has some limitations. The effect of PF4 expression in the macrophages on the cardiac inflammation and RV remodeling needs to be further verified in mouse models. It should also be considered to design small-molecule inhibitors for targeted intervention against PF4 in cardiac macrophages. Besides, novel measurements on RV remodeling events reported by recent studies, including fibrosis, stiffening, and myofiber reorientation, had better be detected in future study.^41^, ^42^, ^43^

In conclusion, our study demonstrated that intense light effectively mitigated the activation and inflammation of specific resident macrophages, thereby attenuating cardiac inflammation and improving hypoxia-induced RV remodeling and dysfunction. Targeting PF4^+^ macrophages in the RVes could represent a novel preventive strategy, while intense light holds promise as an innovative approach for addressing hypoxia-induced RV remodeling and dysfunction.

## CRediT authorship contribution statement

**Dingyuan Tian:** Writing – original draft, Investigation, Data curation, Methodology, Funding acquisition. **Yingzi Pan:** Investigation, Data curation. **Xiaoyue Lai:** Investigation. **Xinyu Bao:** Investigation. **Pan Zheng:** Investigation. **Yan Tan:** Methodology. **Chun Liu:** Validation. **Ziyang Wang:** Investigation. **Qingyuan Yang:** Methodology. **Yang Liu:** Methodology. **Xiaoqin Wan:** Validation. **Zhihui Zhang:** Project administration, Conceptualization. **Fang Deng:** Conceptualization, Funding acquisition, Writing – review & editing.

## Data availability

The data will be made available upon reasonable request.

## Ethics declaration

All experimental procedures were approved by the Animal Ethics Committee of Third Military University (AMUWEC20223610).

## Funding

This work was supported by the Chongqing Natural Science Foundation (China) (No. CSTB2024NSCQ-MSX0645 to D.F.), Army Medical University Science and Technology Innovation Ability Promotion Special Program (China) (No. 2022XQN24 to T.D.Y.), and Key Laboratory Project of Ministry of Education of China (No. PR-KL2022GY009 to T.D.Y.; PR-KL2022GY008 to T.Y.).

## Conflict of interests

These authors declared no conflict of interests.
