# Supplementary material for: Intense light mitigates hypoxia-induced right ventricular remodeling and dysfunction through reducing inflammation associated with PF4+ resident macrophages
Source: Genes Dis. 2025 Oct 21;13(2):101867. doi: 10.1016/j.gendis.2025.101867 (PMC12639265; doi:10.1016/j.gendis.2025.101867)
Supplement: Multimedia component 1 [file mmc1.docx]

**Supplementary Figures**

**
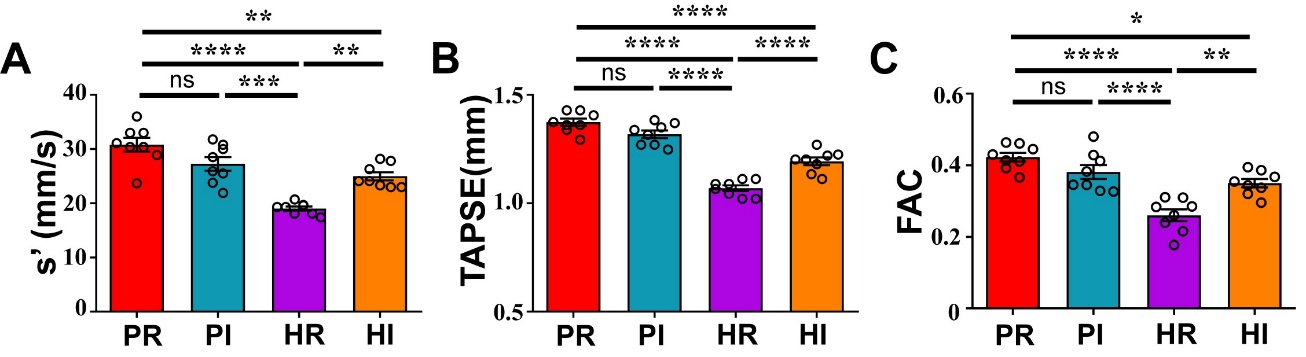
**

**Figure S1.** RV function detected by echocardiographic measurements in each group.

A-C. The s’, TAPSE and FAC levels of mice in each group.

Nomorxia and room light, PR. Nomorxia and intense light, PI. Hypoxia and room light, HR. Hypoxia and intense light, HI. Right ventricular, RV. RV peak systolic myocardial velocity, s'. Tricuspid annular plane systolic excursion, TAPSE. Fractional area change, FAC. n=8 per group. **, *p* < 0.01; *** denotes *p* < 0.001; **** denotes *p* < 0.0001; ns, nonsignificant. The error bars represent the SEMs.

**
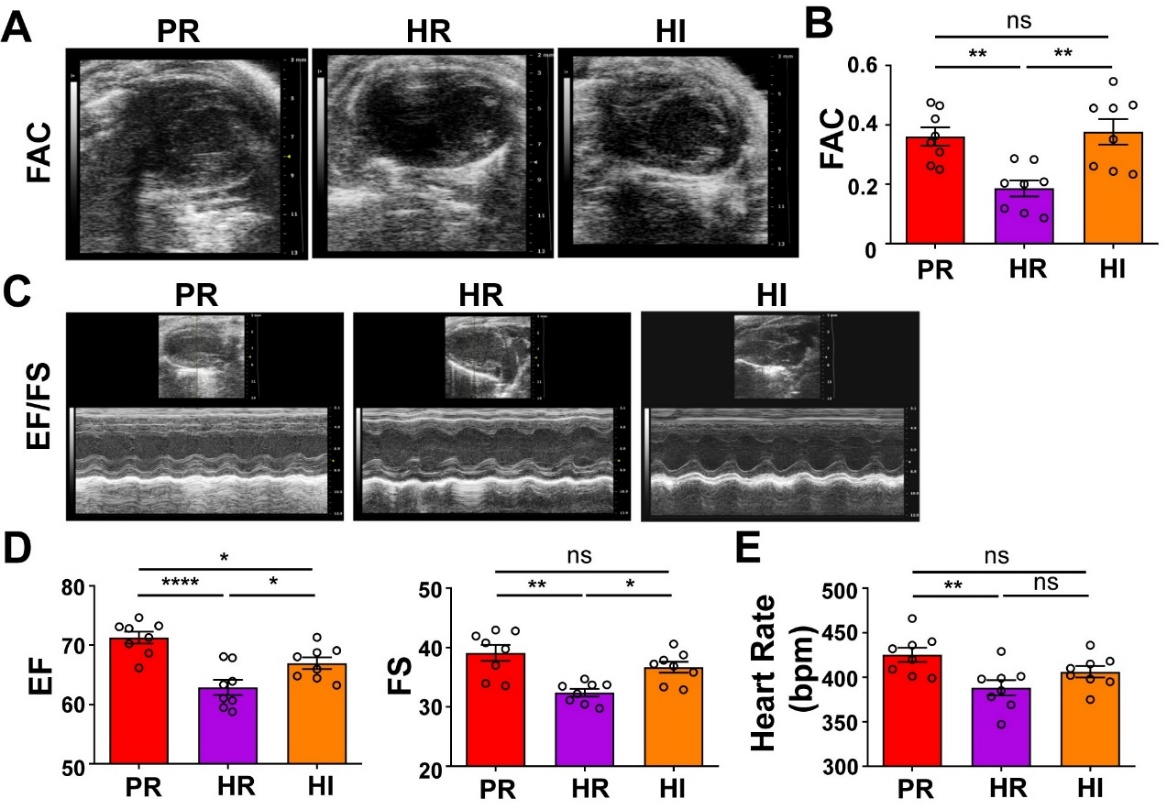
**

**Figure S2.** The levels of LV and RV dysfunction in the mice under normoxia or hypoxia with room light and intense light (PR, HR and HI).

A, B. The FAC level of mice in each group and the statistical analyses.

C, D. The levels of EF and FS of mice and the statistical analyses.

E. The levels of heart rate of mice in each group.

Fractional area change, FAC. ejection fraction, EF. Fractional shortening, FS. Left ventricular, LV. Right ventricular, RV. n=8 per group. *, *p* < 0.05; **, *p* < 0.01; ****, *p* < 0.0001; ns, nonsignificant. The error bars represent the SEMs.


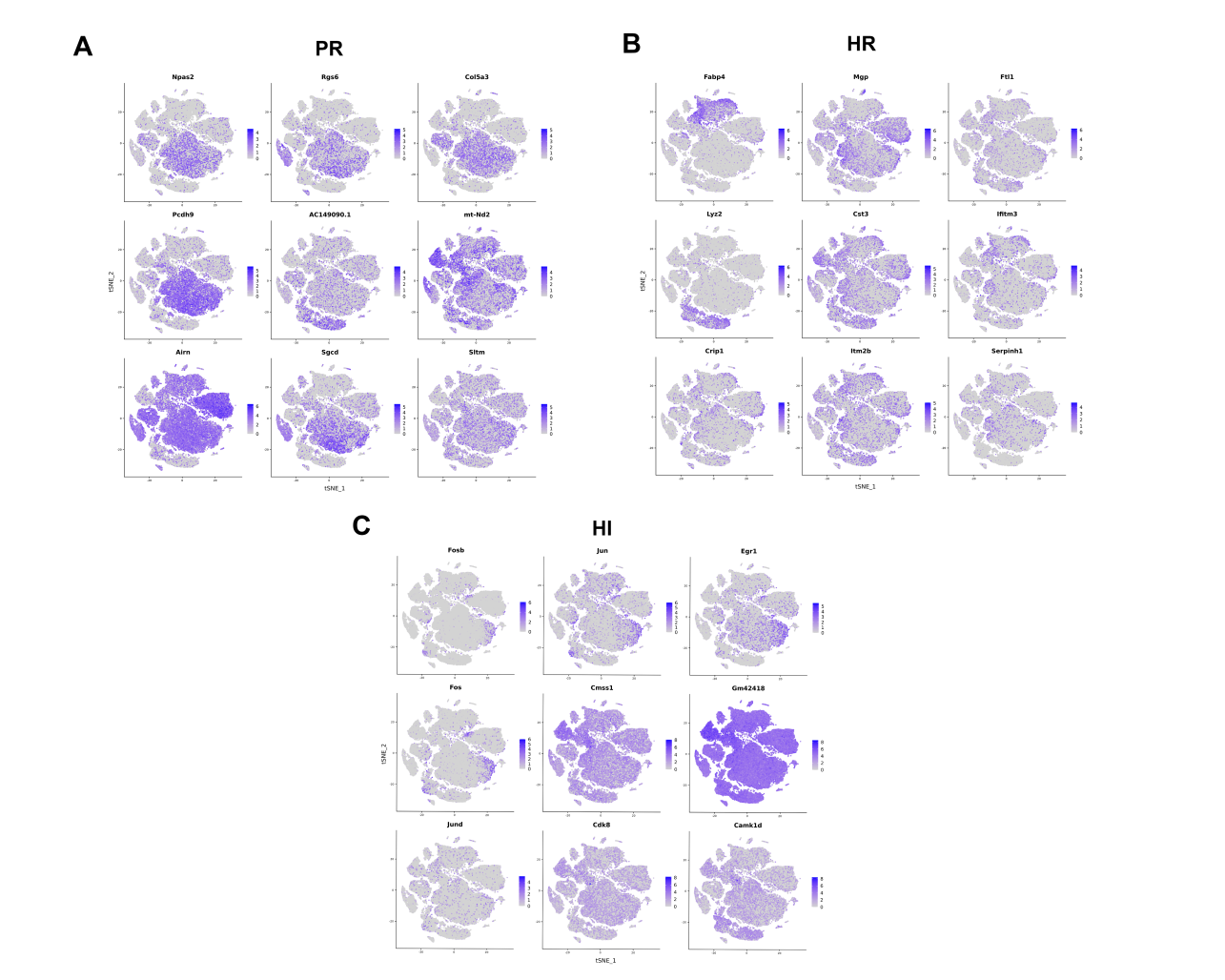


**Figure S3**. Representative genes of the RVes in each group.

The tSNE plots of representative genes identify in PR (A), HR (B) and HI (C) groups. Right ventricles, RVes. n=3 per group.


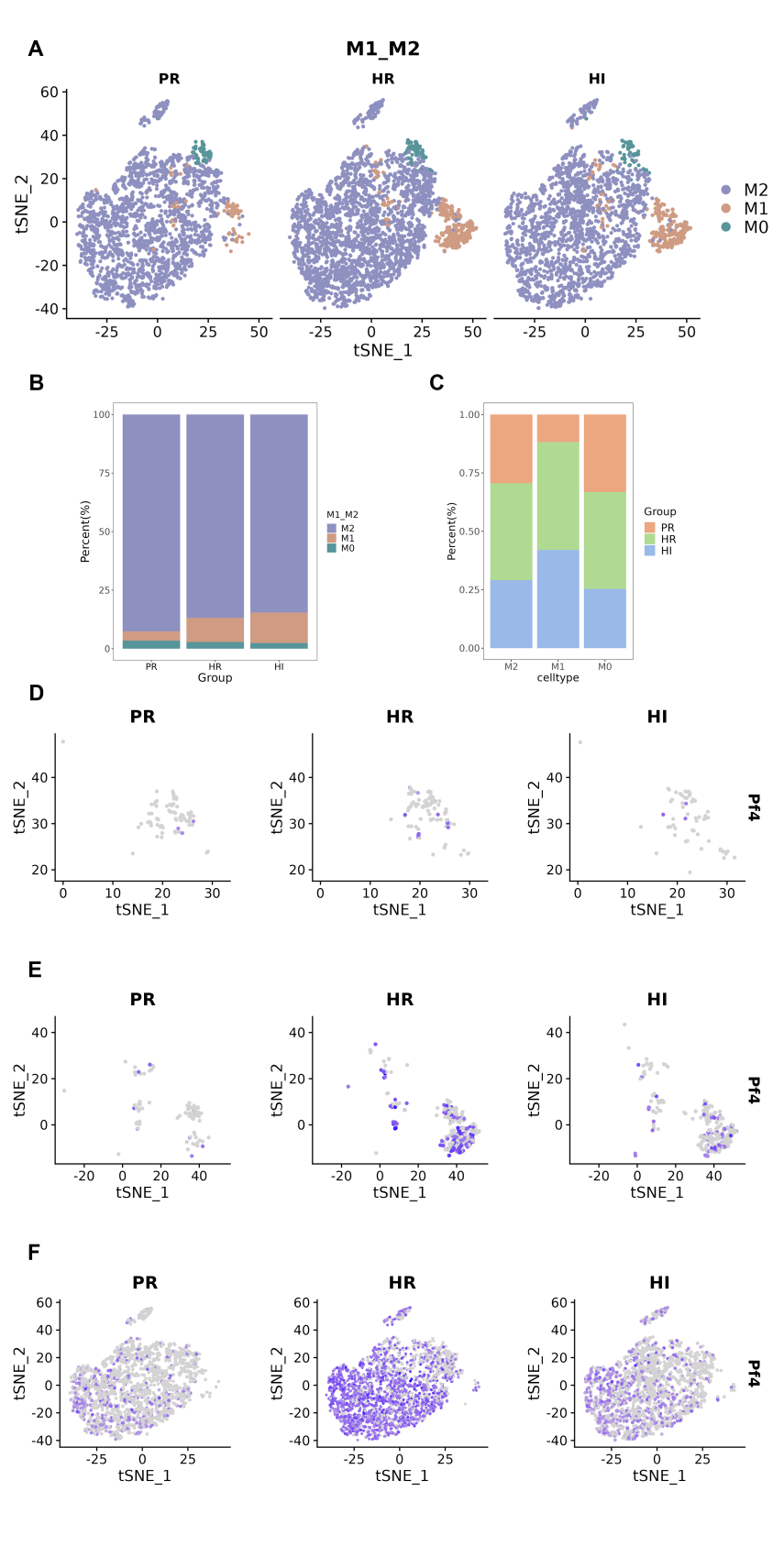


**Figure S4.** Distribution of M0, M1 and M2 macrophages in the RVes of each group.

A. The tSNE plots classified by M0, M1 and M2.

B, C. The proportion of M1, M2 and M0.

D-F. PF4 expression in M1, M2 and M0.

Right ventricles, RVes. n=3 per group.


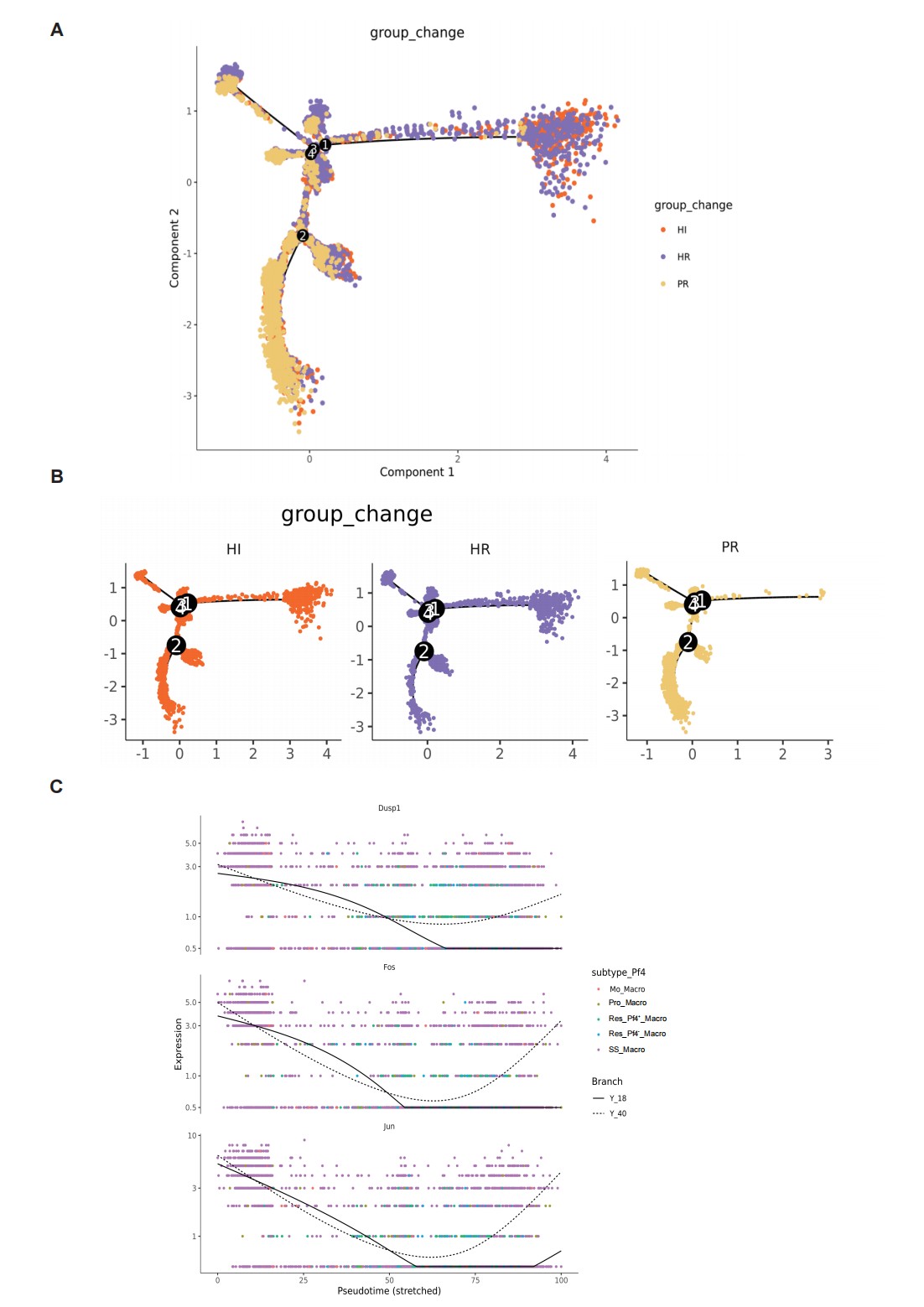


**Figure S5.** The monocle prediction of macrophage subtypes in each group.

A. The monocle prediction in each group.

B. The split monocle prediction in each group.

C. Scatterplot of the "branch 2" node genes. Y_18 represents the directions with small state values, while Y_40 represents the directions with large state values. n=3 per group.

**Supplementary Tables**

**Table S1.** HI vs HR_down and HR vs PR_up genes.

| HI vs HR_down and HR vs PR_up genes |
| --- |
| Serf2 |
| B2m |
| Cst3 |
| S100a6 |
| Rps8 |
| Ndufa4 |
| Mgp |
| Ftl1 |
| Rpl18 |
| Serpinh1 |
| Ifitm2 |
| Ifitm3 |
| Cd81 |
| Dcn |
| Sparc |
| Crip1 |
| Itm2b |
| H2-D1 |
| Fth1 |
| Cox4i1 |
| Cox6a2 |
| Eif1 |
| Rpl37 |
| Lyz2 |
| Fau |
| Cd63 |
| Apoe |
| Gsn |
| Ppib |
| Cox7c |
| Bsg |
| Rpl21 |
| H2-K1 |
| Atp5j |
| Cox6c |
| Atp5g3 |
| Rpl34 |
| Fabp4 |
| Serping1 |
| Rps27a |
| Rps26 |
| Cryab |
| Actb |
| Uqcr11 |
| Rpl9 |
| Tm4sf1 |
| Ly6a |
| Rps24 |
| Clec3b |
| Rpl23 |
| Atp5g1 |
| Cox5b |
| Igfbp7 |
| Ly6e |
| Rpl18a |
| Slc25a4 |
| Col3a1 |
| Rps20 |
| Rps10 |
| Rpl36 |
| Rpl30 |
| Atp5j2 |
| Ctsb |
| Rpl11 |
| P4ha1 |
| Rplp2 |
| Actc1 |
| Fabp3 |
| Atp6v0b |
| Rpl24 |
| Atp5l |
| Tmsb10 |
| Rps4x |
| Atp5e |
| Psap |
| Rplp1 |
| Uqcrq |
| Rps16 |
| Selenop |
| Hsp90b1 |
| Rplp0 |
| Rpl35a |
| Gm11808 |
| Hsp90ab1 |
| Bgn |
| Laptm4a |
| Pdia3 |
| Col1a1 |
| Igfbp4 |
| Rpl13 |
| Crip2 |
| Myl6 |
| Atp5k |
| Rbm39 |
| Hspa8 |
| Tmsb4x |
| Rps28 |
| Rpl8 |
| Timp2 |
| Sec62 |
| Col1a2 |
| Pcolce |
| Rpl19 |
| Tnnc1 |
| Rpl28 |
| Rps2 |
| Rpl41 |
| Eef1a1 |
| Gpx3 |
| Ubb |
| C3 |
| Rpl6 |
| Cd9 |
| Atp5h |
| Actg1 |
| Uqcrh |
| Rpl38 |
| Ctsd |
| Pi16 |
| Ifi203 |
| Calr |
| Calm1 |
| Mat2a |
| Loxl1 |
| Aldoa |
| Cd36 |
| Lamp1 |
| Rpl27a |
| Slc25a3 |
| Rpl17 |
| Rpl37a |
| Fstl1 |
| Tpt1 |
| Mbnl1 |
| Cd47 |
| Rpl5 |
| Rps21 |
| Rps14 |
| Canx |
| Gpx4 |
| Itgb1 |
| Col6a2 |
| Cd34 |
| Col8a1 |
| Dleu2 |

**Table S2.** HI vs HR_down and HR vs PR_up genes.

| HI vs HR_down and HR vs PR_up genes |
| --- |
| Pf4 |
| C1qc |
| C1qb |
| Itm2b |
| Lyz2 |
| Ftl1 |
| Cst3 |
| B2m |
| Apoe |
| H2-D1 |
| C1qa |
| Wfdc17 |
| Ctsc |
| Fth1 |
| Tyrobp |
| Ctsb |
| Malat1 |
| Fcer1g |
| Grn |
| Cd81 |
| Folr2 |
| Cd74 |
| Ly6e |
| Atp6v0b |
| Selenop |
| C5ar1 |
| Ifitm3 |
| H2-K1 |
| Ms4a7 |
| Slc40a1 |
| Rbm39 |
| Ctss |
| Cyba |
| Alox5ap |
| Tmsb4x |
| Tmem176b |
| Cd63 |
| E330020D12Rik |
| Cox4i1 |
| Eif1 |
| Hpgd |
| Ctsd |
| Serf2 |
| Rpl9 |
| Ppib |
| Rps8 |
| Pltp |
| Cd68 |
| Fau |
| Hsp90b1 |
| Rpl34 |
| Crip1 |
| Ifitm2 |
| Hspa5 |
| Mat2a |
| Fcgrt |
| Trps1 |
| Rpl35a |
| Lamp1 |
| Rps11 |
| Rps24 |
| Rpl37 |
| Rps10 |
| Emp3 |
| Ubb |
| Fcrls |

**Table S3.** HI vs HR_down and HR vs PR_up genes.

| HI vs HR_down and HR vs PR_up genes |
| --- |
| Pf4 |
| C1qc |
| C1qb |
| Itm2b |
| Cst3 |
| Lyz2 |
| Ftl1 |
| Malat1 |
| Wfdc17 |
| B2m |
| H2-D1 |
| Fcer1g |
| Apoe |
| Ctsc |
| Fth1 |
| C1qa |
| Tyrobp |
| Ctsb |
| Atp6v0b |
| C5ar1 |
| Cd81 |
| Ly6e |
| Grn |
| Slc40a1 |
| H2-K1 |
| Cyba |
